# Effective pathways for driving self-management among older adults with osteoporosis: a structural equation modeling study based on individual and family self-management theories

**DOI:** 10.3389/fpubh.2026.1877526

**Published:** 2026-07-28

**Authors:** Yuanrong Wang, Wenshu Liu, Xiaofang Liao, Haiyan Wang

**Affiliations:** 1 Department of Geriatric Medical Center, West China Hospital of Sichuan University, Chengdu, China; 2 Department of Nephrology, Sichuan Academy of Medical Sciences and Sichuan Provincial People's Hospital, Chengdu, China; 3 Outpatient Department, Cuqiao Community Health Service Center, Chengdu, China

**Keywords:** older adult osteoporosis, pathway analysis, patient activation, self-management, structural equation model

## Abstract

**Purpose:**

Based on theories of individual and family self-management, this study examines the key factors associated with the activation of self-management among older adults with osteoporosis and the mechanisms underlying these effects, thereby providing a theoretical foundation for developing targeted public health intervention strategies.

**Methods:**

Using a cross-sectional study design, 298 older adult patients with osteoporosis were recruited from a tertiary hospital in Chengdu, China, and the Cuqiao community between June and November 2025. Data were collected using the Patient Activation Measure, the Brief Illness Perception Questionnaire, the Positive and Negative Affect Schedule, the Patient Assessment of Chronic Illness Care, Perceived Social Support Scale, and Osteoporosis Self-Efficacy Scale. Structural equation modeling was employed to validate the predictive relationship between the model variables and patient activation.

**Results:**

Social support and the quality of chronic disease care are the primary factors associated with self-management activation among older adult patients with osteoporosis, exerting both direct and indirect effects; positive emotions and perceived disease threat have no significant direct effects, but both exert indirect effects fully mediated by self-efficacy; negative emotions have both a direct negative effect on self-management engagement and an indirect negative effect by undermining self-efficacy. The model fit is good (CFI = 0.967, RMSEA = 0.025).

**Conclusion:**

Social support and the quality of chronic illness care are key intervention targets that drive self-management activation among older adults with osteoporosis. Efforts should be made to strengthen the family-community-healthcare support network, improve the quality of care services, and enhance patients’ self-efficacy and the early identification of negative emotions to promote active patient participation in health management and improve health outcomes.

## 1 Introduction

Characterized by reduced bone quantity and altered bone microstructure, osteoporosis (OP) increases bone fragility and substantially raises fracture risk (1). The worldwide prevalence of this illness among older adults is 21.7%, and in Asia, it is even higher at 24.3% (2). Therefore, as the global population ages quickly, osteoporosis has become a big public health problem. Therefore, it is not only about bone breaks—the high occurrence rate, caused by disability, and growing medical care expenses, brings a heavy load to families and society. However, managing such a long-term disease cannot be done by doctors alone. Treatment results largely depend on whether patients can maintain self-management behaviors over time (3). But truthfully, most old patients have difficulty in this aspect. Their self-management levels are usually from low to middle, and when following a degree drop, the treatment effect will become worse, which makes them more prone to getting bone fractures (4).

This is the place where “patient activation” enters the picture. It is not merely a predictor of self-management; therefore, it also serves as a standard for measuring patients’ actual level of engagement (5). At its core, patient activation entails patients recognizing their central role in disease management, having the necessary knowledge and skills to manage their health condition, and actively seeking high-quality healthcare services (5). Research confirms that highly patient-activated people adopt healthier lifestyles and achieve better health outcomes (6). Enhancing patient activation among older adults with osteoporosis can therefore yield significant public health benefits. However, despite this great potential, research on the factors that promote patient activation in this specific population remains very limited.

A study has used the Information-Motivation-Behavior model to predict the ability of patients with osteoporosis to engage in self-management behaviors (7), while others have examined how disease-related factors influence health behaviors in patients following a fracture (8). However, most of these studies focus solely on the impact of individual cognitive or biomedical risk factors on self-management among patients with osteoporosis. For older adults who primarily live at home, self-management is not merely a personal matter from a public health perspective; it is a dynamic process influenced by family members and the surrounding community environment. Therefore, there is an urgent need to establish a more comprehensive framework that integrates both individual and social dimensions. The Individual and Family Self-Management Theory (IFSMT) provides precisely such a thing, arranging self-management into three mutually linked dimensions: context, process, and outcomes (9). Based on this framework, this study developed a model to investigate the associations of individual, family, and societal factors on self-management activation among older adults with osteoporosis. By employing structural equation modeling, we aim to elucidate the theoretical pathways among these factors, thereby providing a theoretical foundation for developing interventions that target not only individuals but also families and communities.

## 2 Theoretical model and hypotheses

**Figure 1:**
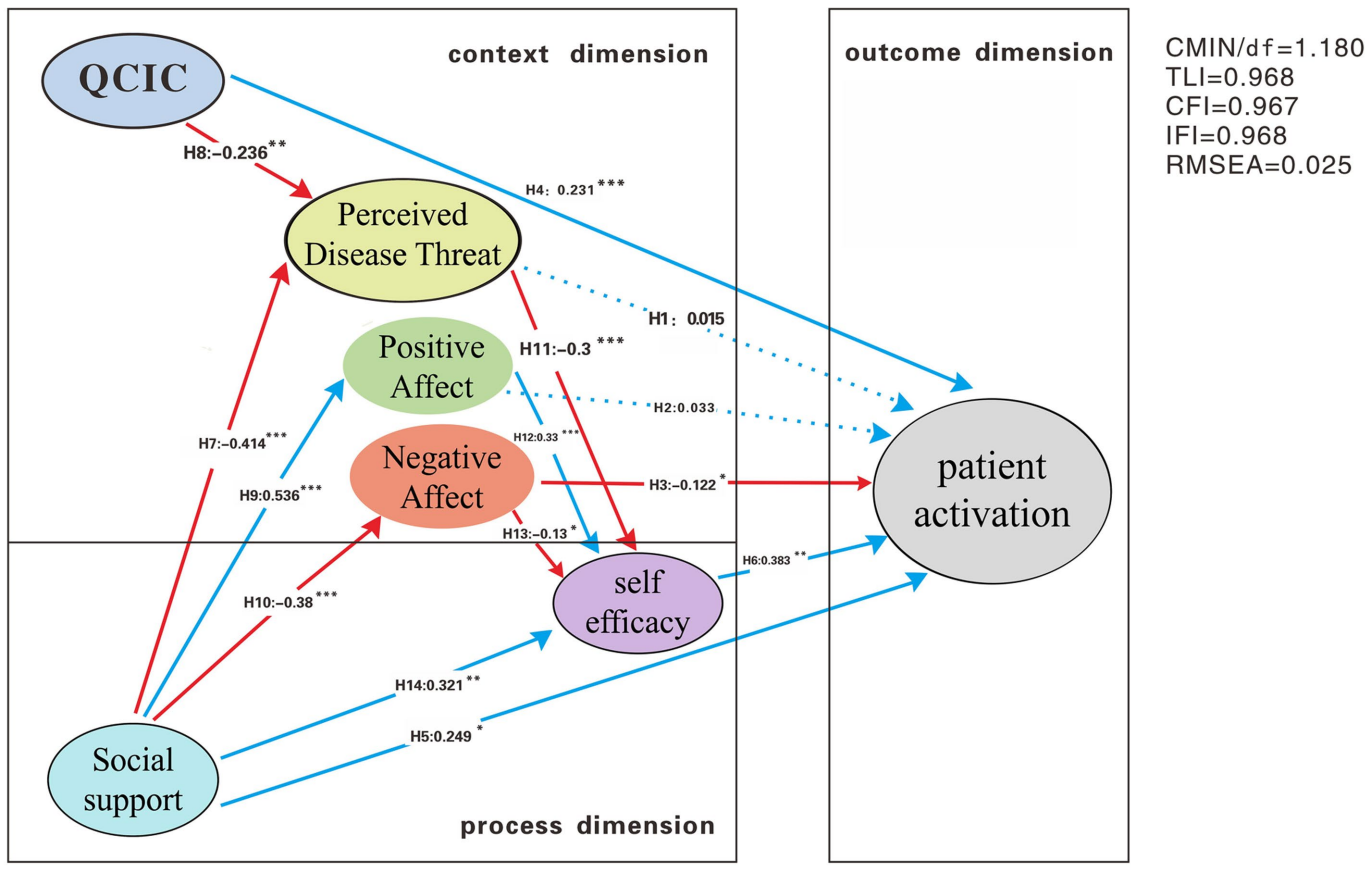
The conceptual framework for the patient activation model in older adult patients with osteoporosis; QCIC, quality of chronic illness care. The structural equation model (SEM) illustrates 14 hypothesized paths and their standardized path coefficients; blue and red arrows indicate positive and negative relationships, respectively. The *R*^2^ value indicates the proportion of variance in the dependent variable explained by the independent variables. Significance levels are denoted by *p* < 0.05 (*), *p* < 0.01 (**), and *p* < 0.001 (***); solid lines indicate that the corresponding path coefficients are statistically significant. Dotted lines indicate that the path is not statistically significant.

At its core, the IFSMT framework regards self-management as dynamic rather than simple—a complex process that unfolds across three interlinked dimensions: context, process, and outcome (9). The context dimension captures the background contexts in which behavior develops, including aspects such as the individual’s disease features and psychological condition, the healthcare settings they navigate, and their broader personal and family circumstances. Therefore, there is a process dimension that touches on the real driving mechanisms of self-management. This relates to the actual efforts people make to achieve their health goals, whether through self-regulatory abilities or through the social support and facilitating factors around them. Hence, the outcome dimension refers to results—what these efforts produce. In this research work, patient activation is the key element we consider: it is the core outcome we focus on, and it also serves as an indicator of the actual degree of patients’ engagement in self-health management. By combining the IFSMT theoretical framework with an analysis of the behavioral features of older adult patients with osteoporosis, in the process of model development, we placed particular emphasis on the malleable and adjustable contextual and process factors within family and social environments, as these factors can directly provide a theoretical basis for the design of interventions. For the contextual dimension, perceived disease threat, positive affect, negative affect, and the quality of chronic illness care were selected as core variables that directly reflect patients’ internal psychological states and their evaluations of the external care environment; for the process dimension, social support and self-efficacy were selected as core variables. Based on theoretical foundations and a literature review, this study proposes 14 basic hypothesis pathways to construct a structural model of factors associated with activation levels in older adult patients with osteoporosis. (Figure 1).

### 2.1 Association between contextual dimensions and patient activation

Health management motivation is directly undermined by negative illness perceptions (10); emotions, as direct carriers of intrinsic motivation, can encourage health behaviors through positive feelings (11); from a social-environmental perspective, professional support from healthcare providers can affect patient activation (12). Therefore, we hypothesize that:

*H1*: Perceived disease threat has a direct, negative predictive association with patient activation.

*H2*: Positive affect has a direct, positive predictive association with patient activation.

*H3*: Negative affect has a direct, negative predictive association with patient activation.

*H4*: Quality of chronic illness care has a direct, positive predictive association with patient activation.

### 2.2 Association between process dimensions and patient activation

Research by Guan et al. confirms that social support exerts an indirect positive influence on patient motivation through mediating factors and serves as a key resource for promoting changes in patient motivation (13). Believing they can affect their disease trajectory, highly self-efficacious patients participate more actively in self-management (14). Therefore, we hypothesize that:

*H5*: Social support has a direct, positive predictive association with patient activation.

*H6*: Self-efficacy has a direct, positive predictive association with patient activation.

### 2.3 Mediating effects of perceived disease threat

Higher social support among older adults with chronic heart failure is linked to fewer negative views of their illness (15). High-quality chronic illness care can help patients develop the belief that their condition is manageable by systematically conveying authoritative information about the disease and sharing successful management strategies, thereby altering their perception of the threat it poses. Therefore, we hypothesize that:

*H7*: Social support has a negative indirect effect on patient activation through the perceived disease threat.

*H8*: The quality of chronic illness care has a negative indirect effect on patient activation through the perceived disease threat.

### 2.4 Mediating effects of positive/negative affect

Social support is a key psychosocial mechanism in moderating patients’ negative illness perceptions. A survey by Chu et al. of 904 older adults in Chinese communities showed that strong social support provides emotional comfort and material assistance, helping them cope with life challenges and reducing negative emotions (16). Therefore, we hypothesize that:

*H9*: Social support has a positive indirect effect on patient activation through the positive affect.

*H10*: Social support has a negative indirect effect on patient activation through the negative affect.

### 2.5 Mediating effects of self-efficacy

Research confirms that among COPD patients, a greater perceived disease threat predicts lower treatment adherence confidence (17); emotional regulation is a key mechanism for enhancing or diminishing self-efficacy, and it functions by buffering or mitigating the impact of stress on behavior (18); Social support is not only a practical resource but also enables individuals to gain a sense of worth, which in turn translates into a belief in their own efficacy. Therefore, we hypothesize that:

*H11*: Perceived disease threat has a negative indirect effect on patient activation through self-efficacy.

*H12*: Positive affect has a positive indirect effect on patient activation through self-efficacy.

*H13*: Negative affect has a negative indirect effect on patient activation through self-efficacy.

*H14*: Social support has a positive indirect effect on patient activation through self-efficacy.

## 3 The study

### 3.1 Study design and participants

From June to November 2025, we conducted a cross-sectional study at a tertiary hospital in Chengdu, China, and within the Cuqiao community, recruiting participants aged 60 years and older with primary osteoporosis. Written informed consent was required for inclusion. Exclusion criteria included severe comorbidities that interfered with participation, cognitive/mental impairments that prevented independent completion of the questionnaire, or refusal to participate. According to numerous studies in the academic community, research involving structural equation modeling should include a sample size of at least *n* = 200 individuals to ensure the reliability of the results (19). Given an expected dropout rate of 10%, the target sample size should exceed 220 cases.

### 3.2 Measures

#### 3.2.1 General characteristics

We recorded participants’ gender, age, highest level of education, living environment, smoking history, drinking history, and history of fractures, which were collected via a questionnaire.

#### 3.2.2 Model indicator system construction

Based on a literature review, this study selected several questionnaires designed by other scholars as representative quantifiable observational variables to construct a model indicator system. The numbers in parentheses indicate the Cronbach’s alpha for the scale / the Cronbach’s alpha in this study.

Patient Activation Measure (20) (PAM-13, Cronbach’s *α* 0.87/0.824); Brief Illness Perception Questionnaire (21)(BIPQ, Cronbach’s *α* 0.65/0.872); Positive and Negative Affect Schedule (22)(PANAS; positive/negative Cronbach’s *α* 0.896/0.866, 0.938/0.950); The Patient Assessment of Chronic Illness Care (23) (PACIC; Cronbach’s *α* 0.93/0.833); Perceived Social Support Scale (24) (PSSS, Cronbach’s α 0.922/0.867); Osteoporosis Self-Efficacy Scale (25)(OSES, Cronbach’s α 0.93/0.861).

### 3.3 Data collection

This study primarily collected data using paper questionnaires. Interviewers assisted participants who had difficulty completing the forms, and the questionnaires were checked immediately upon completion. A total of 306 questionnaires were distributed and collected. To guarantee sample quality, rigorous data screening followed Hair et al.’s methodological guidelines (26): invalid questionnaires completed within 100 s were excluded, and validity was verified through reverse scoring of specific items; additionally, the scales were checked for blank entries, missing responses, or unrecognizable records. Ultimately, 8 invalid questionnaires were excluded through this screening process. Given the small number of missing samples and the requirement for complete data on all observed variables in structural equation modeling, this study adopted the “case deletion” method to handle missing data. Specifically, the 8 questionnaires with missing items were excluded entirely, without replacing missing values with the mean or using any other imputation method. The remaining 298 valid questionnaires were used for subsequent statistical analysis. After handling missing data, normality and outlier tests were conducted to assess the sample distribution and identify potential multicollinearity.

### 3.4 Data analysis

This study used SPSS 26.0 software for descriptive statistics and univariate normality tests, and Amos 24.0 software for model analysis. First, normality and outlier tests were conducted. Then, reliability and validity analyses of the valid questionnaire data were performed using Cronbach’s alpha, CITC analysis, Bartlett’s test, and an assessment of common method bias. To mitigate common method bias, this study implemented several measures: selecting scales with high reliability and validity, ensuring anonymous responses, controlling the measurement time points, and reverse-scoring specific items. The Harman single-factor test was used to assess common-method bias. Next, correlation analyses and multicollinearity tests were conducted among the variables; reliability and validity tests were performed for each latent variable in the model, and model fit was evaluated to ensure that the analysis paths reflected the true relationships. Finally, an in-depth analysis of the interrelationships among different dimensions was conducted, including systematic validation of the structural model and mediation analysis. The mediation analysis employed nonparametric bootstrap resampling with 5,000 repetitions, and a 95% bias-corrected percentile confidence interval was calculated. When the 95% Bootstrap confidence interval does not include 0, the corresponding effect is considered statistically significant.

## 4 Results

### 4.1 General characteristics

The 298 older adult patients with osteoporosis in this study were aged between 60 and 91 years. The majority were female, with most having a secondary school education; the primary living arrangement was cohabitation with a spouse and/or children. Male participants were more likely to have a history of smoking or alcohol consumption, and one-third of the patients had a history of fractures. The activation scores for different types of older adult patients with osteoporosis are shown in Table 1.

**Table 1:** Comparison of patient activation measure scores among older adult osteoporosis patients with different characteristics (*N* = 298).

Demographics	Classification	Number	Mean±SD	F/t	*P*
Gender	Male	103	49.93 ± 11.48	−8.691	0.000***
	Female	195	59.28 ± 13.44		
Age	60 ~ 69	105	61.28 ± 10.71	49.890	0.000***
	70 ~ 79	99	56.38 ± 12.26		
	≥80	94	39.84 ± 13.97		
The highest level of education	Elementary/below	126	41.47 ± 11.79	55.274	0.000***
	Middle school	145	54.84 ± 12.09		
	Hight school	23	59.16 ± 12.46		
	College/above	4	62.71 ± 10.35		
History of fractures	Yes	98	59.18 ± 13.86	20.982	0.000***
	No	200	57.99 ± 11.99		
Living arrangements	Living with spouse and/or children	224	62.31 ± 9.67	0.159	0.854
	Nursing home	61	58.90 ± 10.56		
	Living alone	13	54.44 ± 11.12		
Smoking history	Yes	87	56.32 ± 13.01	1.453	0.063
	No	211	59.32 ± 12.01		
History of alcohol consumption	Yes	99	54.01 ± 12.60	1.776	0.096
	No	199	59.90 ± 11.56		

***p < 0.001.

### 4.2 Patient activation status

The average PAM-13 score for older adult patients with osteoporosis was 59.09 ± 13.88, indicating an overall activation level of Grade 3. The distribution of activation levels across Grades 1 ~ 4 was as follows: 56 patients (18.8%); 69 patients (23.2%); 153 patients (51.3%); and 20 patients (6.7%).

### 4.3 Assessment of multivariate normality and outlier detection

The results of the univariate normality test showed that the skewness of the observed variables ranged from −0.87 to 0.466, with absolute values all less than 2; the kurtosis ranged from −1.32 to 0.005, with absolute values all less than 7, indicating that the data generally met the requirements for univariate normality. The multivariate kurtosis was 109.674, with a critical ratio of 9.845, suggesting that the data did not fully satisfy the strict assumption of a multivariate normal distribution. Further screening of potential multivariate outliers using the squared Mahalanobis distance revealed that the p1 values for samples 133, 266, and 281 were all less than 0.001, indicating that these observations deviated from the sample center in the joint distribution of all measurement indicators. A review of the raw data revealed no values exceeding the scale’s range, obvious data entry errors, severe missing values, or response patterns lacking practical plausibility; therefore, the aforementioned samples were not excluded based on the statistical screening results, and the model analysis was ultimately conducted using 298 valid questionnaires. Given the degree of multivariate non-normality in the data, this study employed the Bootstrap method to estimate standard errors and confidence intervals for the correlation effects, thereby mitigating the impact of distributional deviations on statistical inference. When the 95% Bootstrap confidence interval does not include 0, the corresponding effect is considered statistically significant.

### 4.4 Analysis of the reliability and validity of questionnaire data

The sample demonstrated strong internal consistency, with an overall Cronbach’s *α* of 0.868 and dimension-specific coefficients ranging from 0.833 to 0.950 (>0.8). Corrected item-total correlations (CITC) ranged from 0.480 to 0.885 (>0.4), confirming robust inter-item correlations and high questionnaire reliability. Validity analysis showed a KMO value of 0.924 (>0.8) and a Bartlett’s sphericity test *p*-value <0.001, confirming that the questionnaire data is suitable for factor analysis. Harman’s single-factor test revealed four factors with eigenvalues >1; the first factor accounted for 27.15% (<40%) of the total variance, indicating no significant common method bias.

### 4.5 Correlation analysis and multicollinearity tests

The correlation coefficients between patient activation and perception of disease threat, positive affect, negative affect, social support, self-efficacy, and quality of chronic illness care were −0.464, 0.482, −0.400, 0.534, 0.597, and 0.510, respectively, and all were statistically significant (*p* < 0.05). The results of the multicollinearity analysis indicate that the VIF values for the variables are 1.507, 1.223, 1.024, 1.433, 1.641, and 1.343, respectively—all of which are less than 3, suggesting that the model does not exhibit severe multicollinearity.

### 4.6 Model reliability and validity analysis

To comprehensively evaluate the reliability and construct validity of the measurement model, this study summarized the number of measurement items, standardized factor loadings, composite reliability (CR), average variance extracted (AVE), and discriminant validity results based on the confirmatory factor analysis of each scale. For scales with multiple latent variables, the square roots of the AVE values for each latent variable were compared with the absolute values of the correlations between latent variables to assess discriminant validity across dimensions; the results are shown in Table 2. In addition, the CMIN/DF value of the original model was 1.180, with IFI (0.968), CFI (0.967), and TLI (0.968) all exceeding 0.9, and RMSEA at 0.025 (<0.08). All dimensions met the criteria of an Average Variance Effect (AVE) > 0.5 and a Coefficient of Reliability (CR) > 0.8. The current measurement model is generally acceptable and can serve as a foundation for subsequent structural model testing.

**Table 2:** Results of the reliability and validity tests for the measurement model.

Exogenous variable	Dimension	Number of Items	Range of STD. estimate	CR	AVE	Discriminant validity
Patient activation	—	13	0.676 ~ 0.777	0.936	0.531	—
Positive and Negative affect	Positive affect	10	0.738 ~ 0.812	0.938	0.602	Good
	Negative affect	10	0.766 ~ 0.842	0.95	0.654	Good
Perceived disease threat	Cognize	5	0.672 ~ 0.994	0.884	0.61	Good
	Mood	2	0.737 ~ 0.886	0.797	0.664	Good
Social support	Family support	4	0.728 ~ 0.842	0.867	0.62	Good
	External support	8	0.759 ~ 0.822	0.937	0.649	Good
Self-efficacy	Exercise	6	0.546 ~ 0.905	0.862	0.52	Good
	Calcium intake	6	0.634 ~ 0.774	0.867	0.523	Good
Quality of chronic illness care	Patient initiative	3	0.781 ~ 0.807	0.833	0.625	Good
	Delivery system design	3	0.781 ~ 0.831	0.854	0.662	Good
	Goal setting	5	0.798 ~ 0.814	0.903	0.651	Good
	Problem solving/continuity	4	0.806 ~ 0.829	0.89	0.67	Good
	Follow-up/coordination	5	0.774 ~ 0.817	0.902	0.648	Good

### 4.7 SEM validation and mediation analysis

The goodness of fit of the structural model, based on the conceptual framework of the research variables, was generally good (Figure 1). Since this study employs a cross-sectional survey design, the unidirectional paths and their path coefficients in the structural equation model are used to test the fit between the variable relationships specified in the theoretical model and the sample data. They reflect the direction and strength of statistical associations between variables under specific model conditions; they do not imply a temporal sequence among the variables, nor can causal inferences be drawn based on them. The results show that, among the 14 path hypotheses, only H1 (*z* = 0.209, *p* = 0.834 > 0.05) and H2 (*z* = 0.511, *p* = 0.609 > 0.05) were not statistically significant; the remaining 12 hypotheses (H3–H14) were all supported, indicating that perceived disease threat and positive emotions are not directly associated with the activation levels of older adult patients with osteoporosis; instead, both are fully mediated by self-efficacy (Table 3). Social support and the quality of chronic disease care had the strongest combined effect on patient activation (Table 4).

**Table 3:** Structural model path analysis results.

Hypothesis	Relationship			Estimate	S. E.	C. R.	P	Std.(*β*)
H1	Perceived disease threat	→	patient activation	0.003	0.015	0.209	0.834	0.015
H2	Positive affect	→	patient activation	0.003	0.005	0.511	0.609	0.033
H3	Negative affect	→	patient activation	−0.009	0.004	−2.495	0.013	−0.122
H4	QCIC	→	patient activation	0.207	0.056	3.675	***	0.231
H5	social support	→	patient activation	0.201	0.088	2.271	0.023	0.249
H6	self-efficacy	→	patient activation	0.161	0.052	3.087	0.002	0.383
H7	social support	→	perceived disease threat	−1.527	0.339	−4.505	***	−0.414
H8	QCIC	→	perceived disease threat	−0.97	0.325	−2.988	0.003	−0.236
H9	social support	→	Positive affect	5.453	0.785	6.947	***	0.536
H10	social support	→	Negative affect	−4.294	0.812	−5.289	***	−0.38
H11	perceived disease threat	→	self-efficacy	−0.156	0.042	−3.711	***	−0.3
H12	Positive affect	→	self-efficacy	0.062	0.013	4.612	***	0.33
H13	Negative affect	→	self-efficacy	−0.022	0.01	−2.248	0.025	−0.13
H14	social support	→	self-efficacy	0.616	0.21	2.929	0.003	0.321

***p < 0.001, QCIC, quality of chronic illness care.

When *p* < 0.05, the path is significant. If a path is significant and the coefficient is positive, the independent variable has a significant positive effect on the dependent variable.

**Table 4:** Standard direct, indirect, and total effects of the patient activation model.

Endogenous variable	Exogenous variable	Total effect			Direct effect			Indirect effect			Total effect ranking
		Estimate	Bootstrap 95% CI		Estimate	Bootstrap 95% CI		Estimate	Bootstrap 95% CI		
			Lower bounds	upper bounds		Lower bounds	upper bounds		Lower bounds	upper bounds	
Patient activation	Self-efficacy	0.168	0.081	0.28	0.168	0.081	0.28	—	—	—	3
	QCIC	0.228	0.109	0.342	0.203	0.094	0.311	0.025	0.006	0.06	2
	social support	0.457	0.285	1.178	0.204	0.012	0.428	0.253	0.046	0.459	1
	perceived disease threat	−0.026	−0.05	−0.01	—	—	—	−0.026	−0.05	−0.01	4
	Positive affect	0.011	0.004	0.018	—	—	—	0.011	0.004	0.018	6
	Negative affect	−0.012	−0.019	−0.005	−0.008	−0.015	−0.002	−0.004	−0.009	−0.001	5

QCIC, quality of chronic illness care.

The 95% confidence intervals do not include zero, indicating that the total effect, direct effect, or mediating effect is significant. The total effect values are also ranked. The table shows the direct and mediating effects of the independent variable on patient engagement. The 95% confidence intervals do not include zero, indicating that the total effect, direct effect, or mediating effect is significant. The total effect values are also ranked.

## 5 Discussion

With a mean score of 59.09 ± 13.88, older adult patients with osteoporosis exhibited moderate levels of patient activation. According to Hibbard et al.’s description of Level 3 activation, older adult patients with osteoporosis have begun to engage in health-promoting behaviors but lack the confidence and skills for sustained health management and have not yet developed stable, self-directed habits (5). This may reflect improvements in chronic disease management and community health education, which have increased osteoporosis awareness among older adults. However, the insidious nature of osteoporosis symptoms (27), the lack of immediate treatment effects (28), and joint discomfort during exercise (29) may lead patients to discontinue or abandon their management plans easily. Therefore, public health strategies should go beyond awareness campaigns and focus on building confidence and behavioral skills. Notably, 18.8% of participants exhibited the lowest level of activation. These patients passively received care and had not yet recognized their central role in managing their own health. Evidence shows that persons with low activation gain the greatest benefits from intervention measures (30). Therefore, community health services can use simple screening tools to identify this high-risk group, which is a low-cost, high-impact public health measure. Additionally, activation scores differed significantly by age, gender, education level, and fracture history. This suggests that targeted interventions should prioritize older women with low educational attainment who have a history of fractures; such precise targeting can maximize health benefits at the population level.

The most striking finding of this study is that social support is most strongly associated with activation among older adults with osteoporosis, both directly and indirectly. This highlights the importance of strengthening family and community support networks in public health practice. Family support—especially the daily care and emotional solace provided by adult children—is the most direct and dependable source of support for older adult patients (31). Meanwhile, outside backing from friends, the same patients, the community, and health care providers provides special advantages in providing various information and professional expertise. Therefore, since osteoporosis management is a long-term process integrated into daily life, and because older adult persons usually have declining cognitive capacities and executive function, external support becomes an irreplaceable pillar when facing difficulties with medication adherence, regular exercise, and dietary habit adjustment. At the same time, social support may also be indirectly associated with patient activation through factors such as the perceived threat of illness, emotional state, and self-efficacy. This may be because when older adults feel cared for by their families and society, their psychological need for care and acceptance is met. This positive mental state helps them feel less afraid of illness and gives them greater confidence to stick with their treatment and exercise routines, thereby encouraging them to take a more active role in managing their health. Therefore, activation interventions for older adult patients with osteoporosis should establish a “family–community–medical” social support network: for patients with weak family support, the community should assume the role of “supplementing family support” by providing continuous emotional and informational support through regular follow-ups; for patients with strong family support, family members should be guided to provide “empowering support,” balancing care and supervision to prevent patients from becoming overly dependent and thereby weakening their self-management abilities.

The structural equation model in this study indicates that the overall association between the quality of chronic illness care and patient activation is second only to that of social support. Tedesco et al. (3) found that interactions with healthcare professionals play a crucial role in the daily care and self-management of patients with osteoporosis. Older adults are characterized by multimorbidity and a high degree of trust in medical authority. High-quality chronic illness care can compensate for deficiencies in self-care capabilities while leveraging trust in medical authority to promote behavioral change. Therefore, from a public health perspective, high-quality chronic disease service networks should be promoted in primary care settings, exploring the “Internet + follow-up” model to enhance service accessibility through mobile health technologies (32), ensuring that older adult patients living alone or with mobility limitations can also receive continuous professional support.

This study shows that, unlike the direct impact of disease perception on self-management among patients with spinal disorders (33), the perception of disease threat is not directly associated with activation in older adult patients with osteoporosis; rather, it acts entirely indirectly through self-efficacy. This finding highlights a mechanism that may have been overlooked in traditional public health education: simply raising older adults’ awareness of osteoporosis risks is not enough. Interventions must also enhance self-efficacy—for example, by helping patients set achievable, incremental goals and providing visual feedback through health apps.

This study also indicates that while positive affect shows no direct, significant association with patient activation, it can exert an indirect positive association by enhancing self-efficacy. Negative affect, on the other hand, exhibits both a direct negative association and an indirect negative association through the reduction of self-efficacy. Therefore, public health programs should incorporate emotional regulation training into routine elder care, teaching caregivers to identify and manage negative emotions and to foster positive emotional experiences by reflecting on past successes. Lao et al. also noted that multi-pathway interventions based on self-efficacy theory are effective strategies for improving patients‘mental health and promoting long-term health management (34).

Despite the above findings, this study has several limitations. This study employed a cross-sectional design, and all path analyses were conducted as empirical tests of a theoretical model. Furthermore, due to resource constraints, the sample was drawn exclusively from a specific region, which may introduce regional bias; additionally, the study did not include objective geriatric-specific indicators such as bone mineral density, fracture severity, frailty indices, cognitive function, or polypharmacy, nor did it utilize multi-source data (e.g., family reports, medical records). Consequently, the risk of common-method bias cannot be entirely ruled out. These limitations suggest that future studies should employ a longitudinal design, utilize multicenter samples, and incorporate objective clinical indicators to further validate and expand upon the findings of this study.

## 6 Conclusion

The study findings indicate that the activation level among older adult patients with osteoporosis is at Level 3. This suggests that their self-management capacity is moderate but still has room for improvement. Based on the IFSMT theory, this study reveals that social support and the quality of chronic disease care are the two core factors most strongly associated with the overall self-management activation of older adult patients with osteoporosis. Perceived disease threat and positive emotions show no direct statistical association; instead, they exert an indirect effect on patient activation, mediated by self-efficacy. The findings provide potential intervention targets for policymakers and primary care providers: for primary public health and clinical interventions, priority should be given to establishing a “family–community—primary care” multi-dimensional social support system and continuously optimize primary care services such as online remote care for osteoporosis; the focus of clinical communication and health education should shift from merely disseminating information about disease risks to enhancing self-efficacy; routine screening of older adults with low activation should be implemented; early identification of negative emotions and psychological counseling for older adults should be conducted simultaneously; and the intrinsic motivation for self-management among older adults with osteoporosis should be stimulated from multiple dimensions, ultimately improving long-term skeletal health outcomes and enhancing quality of life.

## Data Availability

The raw data supporting the conclusions of this article will be made available by the authors, without undue reservation.
